# Editorial: Emerging Concepts of Innate Immune Responses to Neglected Tropical Diseases

**DOI:** 10.3389/fimmu.2021.658553

**Published:** 2021-09-15

**Authors:** Malcolm S. Duthie, Yasuyuki Goto

**Affiliations:** ^1^Research and Development, HDT Bio, Seattle, WA, United States; ^2^Department of Animal Resource Sciences, Graduate School of Agricultural and Life Sciences, The University of Tokyo, Tokyo, Japan

**Keywords:** innate, infection, skin, spleen, neglected tropical disease (NTD), lymph node

Neglected tropical diseases (NTDs), a collective of infectious diseases predominantly reported in under-resourced settings, directly affect over a billion people. Multiple initiatives have been launched to combat NTDs but they continue to place a significant economic and social burden on developing nations. Beyond their immediate importance, studies of NTDs have become important pillars of immunology: clinical observation, and experimental animal models, of NTDs helped define the Th1/2 paradigm (1–3). This Research Topic, presented as original research and review articles, focused on recent advances in our understanding of innate immune mechanisms and cell subsets, and of how innate cells can influence the outcome of NTDs.

Van Bockstal et al. demonstrated that type I interferons exacerbate *Leishmania* infections associated with visceral disease. Enhanced macrophage susceptibility to *Leishmania infantum* and *L. donovani* following incubation with IFN‐α was linked to upregulated sialoadhesin (Siglec-1/CD169, Sn) surface expression and in a series of experiments involving Sn‐deficiency or blockade, susceptibility was restored to normal levels. These data raise the possibility that targeting of Sn in early *Leishmania* infection could limit progression to visceral disease.

Lecoeur et al. applied an intricate flow cytometry-based procedure to demonstrate that *Leishmania* subverts the transcription factor landscape by escaping the Toll-like receptor (TLR)–NF-kB–NLRP3 axis and stalling maturation. Transcriptomic analyses of *Leishmania*-infected DC revealed that DC infected by non-opsonized parasites maintained an immature phenotype that was associated with down-regulation of genes related to pro-inflammatory TLR signaling while infection of DC with opsonized parasites enhanced this profile, with DC displaying a semi-mature phenotype. The authors reasonably speculate that this *Leishmania*-specific signature could have relevance to infection with other intracellular pathogens.

Although myeloid innate immune cells are the dominant sensors of microbes, Stögerer and Stäger highlight that B and T cells also express PRRs like TLRs. TLR signaling, in particular MyD88-dependent TLRs and endosomal TLR7 and TLR9, is involved in autoreactive B cell activation and in visceral leishmaniasis is proposed to cause hypergammaglobulinemia and disease exacerbation. DAMPs can trigger TLR7 and induce cell death in T cells during chronic infection of *Leishmania donovani*. It is thus important to consider the effect of TLR agonists on T and B cells, and not only on myeloid cells, when developing new vaccination strategies, particularly for therapeutic purposes.

The disruption of splenic white pulp is accompanied by disease progression in mammals that are susceptible to fatal visceral leishmaniasis. De Melo et al. document morphological remodeling of splenic compartments following experimental *L. infantum* infection. One month after parasite inoculation of BALB/c mice a decline in the number of plasmacytoid DC was observed along with hyperplasia in the white pulp. By two months, splenomegaly remained and was now associated with increased numbers of macrophages, B and T cells and plasma cells. Unlike the organized distribution of lymphoid tissue inducer (LTi) cells around the periarteriolar lymphoid sheath observed in control mice, LTi were increased and scattered throughout the red pulp in *Leishmania*-infected mice. By three months of infection, the relative frequencies of follicular and plasmacytoid DC were increased and splenic IL-6 and IFN-gamma production had commenced.

In their contribution Serrano-Coll et al. speculate that alterations in the skin and peripheral nerves during leprosy are related to activity in the Notch signaling pathway. They observed that *Hes-1* gene expression was downregulated, while *Runx-1* was upregulated, in the skin of leprosy patients. Immunohistochemistry revealed a corresponding reduction of Hes-1 protein levels in the epidermis, eccrine glands and hair follicles. Increased expression of Runx-1 was observed in infiltrating inflammatory cells. The authors reason that these changes could render the infected skin conducive for *M. leprae* survival and proliferation.

While *Mycobacterium leprae* causes leprosy, the vast majority of exposed individuals do not develop disease. Van Hooij et al. assessed the relationship between innate immune markers and *M. leprae* infection by studying individuals at high risk of infection. Similar to leprosy patients, the individuals at risk had higher ApoA1 and S100A12 levels than control subjects. Importantly, higher S100A12 and lower CCL4 levels in response to *M. leprae* were observed among households where *M. leprae* infection and leprosy were not detected beyond the index case, suggesting that these molecules are involved in a response that restricts *M. leprae* dissemination.

Boldt et al. took a unique retrospective approach of comparing leprosy patients who have had/had not HBV infection and addressed associations of genes in the complement pathway with susceptibility to HBV in leprosy patients, demonstrating polymorphisms compromising activation of the lectin pathway of complement, especially ficolin, as modulating the susceptibility. The associations suggest a critical role of the lectin pathway in controlling HBV infection which is maintained and even reinforced within the context of leprosy disease. Thus, residents in NTD-endemic areas can experience altered responses against other immune challenges, posing obvious and significant public health challenges.

While most pathogenic mycobacteria evade innate immune responses to replicate inside host macrophages and induce granulomas that contain but do not eliminate the bacteria, *Mycobacterium ulcerans* has acquired the virulence plasmid pMUM that allows the synthesis of mycolactone, a macrolide toxin that causes the chronic, necrotizing skin lesions that characterize Buruli ulcer. By comparing the pathogenesis of *M. ulcerans* and *M. marinum* diseases, Röltgen and Pluschke summarize current data on innate immune mechanisms against *M. ulcerans* infection. They note that Bacillus Calmette-Guérin (BCG) has limited short-term protective activity against Buruli ulcer and conclude that a more detailed understanding of innate immunity to *M. ulcerans* infection is required to develop an effective vaccine for control in endemic areas.

While the pathogenesis of experimental trypanosome infections has been widely studied after intraperitoneal or intravenous injection, real world infection with African trypanosomes begins when the tsetse fly vector injects the parasites into the dermis during blood feeding. In their review article, Alfituri et al. highlight recent studies of initial infection in the skin. They suggest that by thoroughly defining the mechanisms involved in both establishing African trypanosome infections in the skin and in facilitating their progression through the host, novel approaches to control may be revealed. By assessing *Trypanosoma brucei* infection in lymphotoxin‐β‐deficient mice (LTβ‐/‐ mice), the group tested their hypothesis that the absence of the skin draining lymph nodes would impede the establishment of infection (Alfituri et al.). To their surprise, LTβ‐/‐ mice exhibited greater susceptibility to *T. brucei* infection, indicating that the early accumulation of the trypanosomes in the skin draining lymph nodes was not essential for systemic infection. Restoration of the microarchitecture of the B cell follicles in the spleens of LTβ‐/‐ mice reduced susceptibility to intradermal *T. brucei* infection and IgG class-switched parasite-specific antibodies became apparent in the circulation. These data suggest that organized splenic microarchitecture is essential for the control of African trypanosomes.

Magez et al. review how innate immune components control salivarian trypanosomes. To avoid their overpopulation and killing of the host, trypanosomes have acquired a system of quorum sensing for density-dependent population growth arrest. The same system could possibly sense infection-associated host tissue damage, in which case the quorum sensing serves to prevent excessive immunopathology. The researchers’ own experiments showed that vaccine-induced immune modulation of inflammation, and the CD1d molecule, was central to protection during trypanosome infection. They extend their review by discussing reports indicating that trypanosomes compromise the general host immune system so as to cause significant reductions in the efficacy of multiple non-trypanosome related vaccines (Magez et al.). In a similar vein, Osii et al. reviewed current understanding of how *Plasmodium* infection disrupts DC-T cell interactions to generate T cells that fail to help B cell responses. This not only reduces the production of antibodies necessary to control malaria, but by upregulating negative regulatory molecules *Plasmodium* infection also induces CD4 T cell exhaustion.

*Schistosoma* parasites enact numerous strategies, including the production of immunomodulatory molecules, alteration of membranes and the formation of granulomas, to promote infection without detrimental exposure to the immune response (Angeles et al.). Keratinocytes, macrophages, DCs and B1 cells produce IL-10 in the skin in response to the cercarial invasion and Sm16, a major molecule from the secreted protein of the cercaria can prevent classical activation macrophage and delay antigen processing. Excretory-secretory products from the cercaria and schistosomula also induce inhibitory molecules such as prostaglandins.

Obata-Ninomiya et al. detail that helminths interact with various epithelial cell surfaces, with the cells subsequently producing a series of type 2 epithelial cytokines that lead to the induction of innate and acquired type 2 immune responses. Increased frequencies of basophils and eosinophils have been documented during helminth infections and in response to nematode parasite re-infection, basophils accumulate in the peripheral tissues for IgE release and secretion of proteases that recruit monocytes, neutrophils and eosinophils. In response to IL-5, eosinophils produce major basic protein to kill the parasites.

By characterizing innate responses in experimental models, and observing them in clinical or veterinary settings, of NTDs, valuable insight is being generated for the development of new control strategies (diagnostics, immune therapy, enhanced vaccination). With the realization of similarities with other infectious and non-infectious diseases, this knowledge can have significant impact beyond NTDs.

## Author Contributions

MD and YG wrote this editorial. All authors contributed to the article and approved the submitted version.

## Funding

The authors of this editorial are funded by The Global Health Innovative Technology Fund (G2018–111). NTD research conducted by MD has also been funded by grants from CRDF Global (#62966), National Institute of Allergy and Infectious Diseases of the National Institutes of Health under Award Number R01AI025038 and R21AI144641, and the Bill and Melinda Gates Foundation (#631 and #39129), American Leprosy Missions, Leprosy Research Initiative, and de Turing Foundation. NTD research conducted by YG has been supported by Japanese Society for the Promotion of Science grants (#18H02649 and #20K21516), Japan Agency for Medical Research and Development grant for Translational Research Network Program grant Seed A and Japan Agency for Medical Research and Development grant for U.S.–Japan Cooperative Medical Sciences Program.

## Author Disclaimer

The content is solely the responsibility of the authors and does not necessarily represent the official views of the funding bodies.

## Conflict of Interest

Author MD was employed by the company HDT Bio.

The remaining author declares that the research was conducted in the absence of any commercial or financial relationships that could be construed as a potential conflict of interest.

## Publisher’s Note

All claims expressed in this article are solely those of the authors and do not necessarily represent those of their affiliated organizations, or those of the publisher, the editors and the reviewers. Any product that may be evaluated in this article, or claim that may be made by its manufacturer, is not guaranteed or endorsed by the publisher.
